# Double nanowire quantum dots and machine learning

**DOI:** 10.1038/s41598-025-89443-w

**Published:** 2025-02-18

**Authors:** Michał Zieliński

**Affiliations:** Institute of Physics, Faculty of Physics, Astronomy and Informatics, Nicolaus Copernicus University in Toruń, Toruń, Poland

**Keywords:** Double quantum dots, Nanowire quantum dots, Machine learning, Deep learning, Quantum dots, Information theory and computation

## Abstract

We present an approach to estimate the single-particle energies in double InAs/InP nanowire quantum dots by combining an atomistic tight-binding approach with machine learning. The method works particularly well with a neural network and transfer learning, where we can accurately recover ground state energies with root-mean-square deviation around 1 meV by using only a small training set and capitalizing on earlier, smaller-scale computations. The training set is only a fraction of the multidimensional search space of possible dot sizes and inter-dot spacings. Besides the cases presented in this work, we expect this technique will interest other researchers involved in solving the inverse computational problem of matching spectra to nanostructure morphological properties.

## Introduction

Nanowire quantum dots^1–5^ give the benefit of effective spatial positioning, combined with high-quality optical spectra.^6–9^ Besides single dots, nanowires can host double or multiple quantum dots,^10–13^ whereas coupled quantum dots can be considered a solid-state counterpart of molecules.^14–21^ The computation of spectral properties of nanowire quantum dots presents a formidable computation challenge. Often, time-consuming atomistic calculations^22,23^ must be used, as they account for sharp interfaces,^24^, alloying,^4,25^ or substrate orientation.^26^ These calculations may involve millions of atoms in the simulation, presenting a formidable computational challenge even for a single system. Double quantum dots can have different inter-dot separations, each of the dots may have a different shape (e.g. lens, disk, pyramid), different size (e.g. radius, diameter),^14–17,19–21^ as well as different chemical composition.^27–31^ Dots can be elongated along different crystal axes,^32–34^ misaligned^35,36^to each other, or there may be pronounced alloy randomness.^27–31^ All these lead to a very large number of possible systems, forming a multidimensional (search) space. Typically, this problem is omitted by setting most of these (hyper-)parameters to some reasonable values (especially if there is an input from the experiment) and limiting the double quantum dot spectra calculation to a 1D plot, typically as a function of the inter-dot separation. Whereas such an approach has its merit, particularly in addressing a specific experiment, it does not necessarily offer a predictive capability as the researcher cannot (due to complexity) compute spectral properties over the large (hyper-)space of possible double dot morphologies. In this work, we present an approach that may be a step toward this direction by utilizing a machine learning approach,^37,38^ particularly neural networks.^39^ These have stormed quantum physics and chemistry with multiple possible applications in recent years.^40–44^ In this paper, we test several different machine learning schemes that, in principle, could allow us to reduce computational burden significantly – or conversely – perform systematic, multi-dimensional space search by performing time-consuming calculations on only a relatively small subset of all possible double dot geometries, and predict properties of all other systems via machine learning.

## Results

**Fig. 1:**
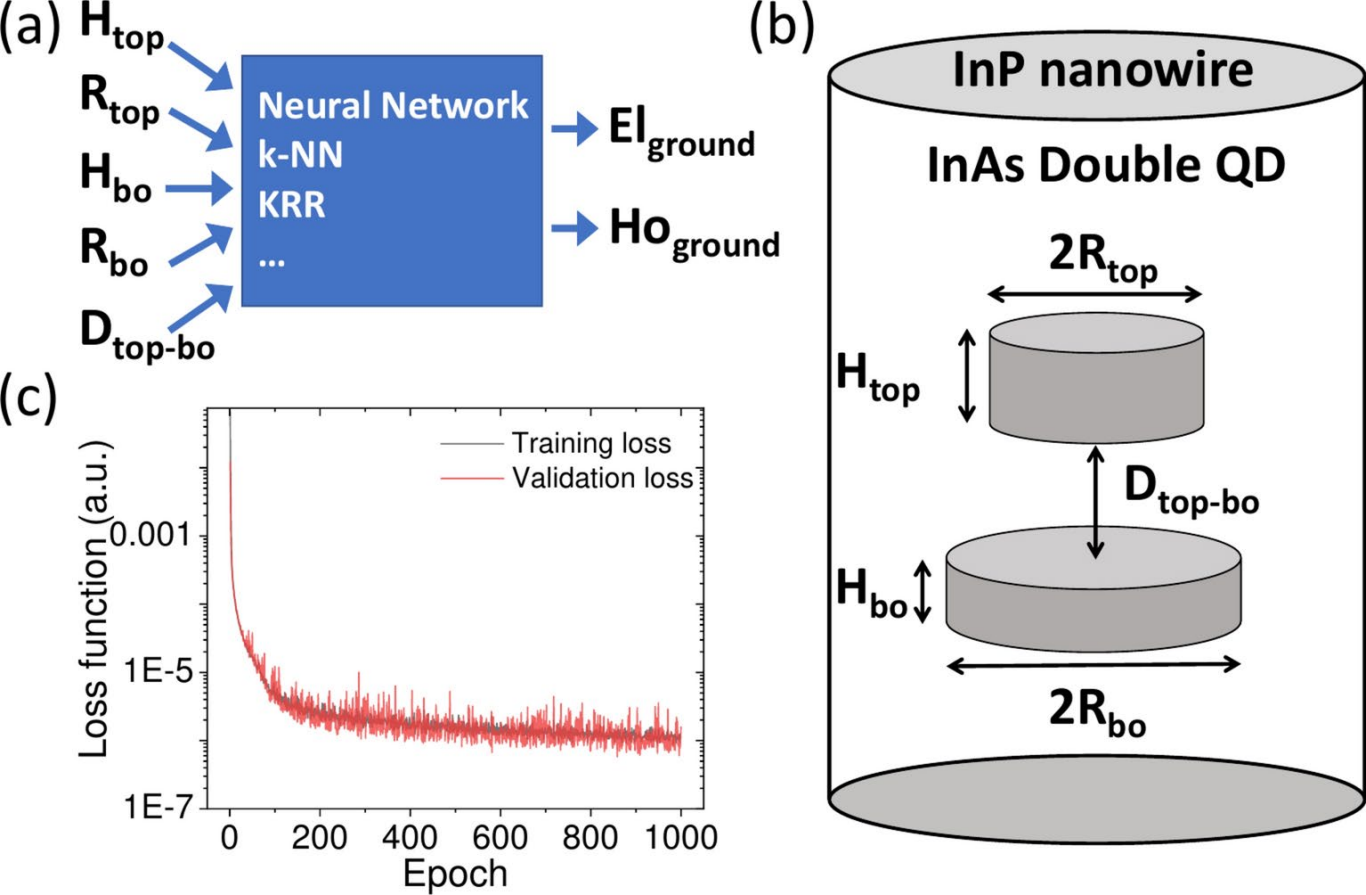
Schematics of (**a**) computational ”black box” approach, (**b**) physical systems under consideration – double InAs/InP nanowire quantum dot of different sizes and inter-dot ($D_{lo-up}$) separation, and (**c**) an exemplary plot showing the training process (using the tight-binding data) with training and validation loss during following epochs (note the logarithmic scale on the vertical axis).

This work aims to establish a computational scheme that will take double quantum dot morphological parameters (”features”) and produce an output (”labels”) in terms of their spectral properties. Here, due to practical reasons, we limit ourselves to five input features (5-D search space), i.e., the height of the top and bottom quantum dots ($H_{top}$, $H_{bo}$), their radii ($R_{top}$, $R_{bo}$), and the inter-dot separation ($D_{top-bo}$), as shown schematically in Fig. 1 (a,b). The labels, or output variables, are the electron ground and hole energies ($El_{ground}=E1$, $Ho_{ground}=H1$). Many other possible features (e.g., axial dot misalignment or composition intermixing) could be included; however, we leave that for our future work. Moreover, multiple other output variables, such as excited states^44^ energies or excitonic complexes spectra, could be considered here. Again, we leave this to our future work.

In atomistic calculation, as well as in the experiment, due to the presence of the underlying crystal lattice, dot dimensions and separation can be expressed only in discreet dimensions (monolayer and its multiples); here, we limit ourselves to a unit of a lattice constant (l.c.). Further, in a practical calculation, we must limit ranges of possible feature values, which for dot heights, we set to vary as $H=$1,2,3,4,5; for dot radii $R=6,7,8,9,10$, and for the inter-dot separation $D_{top-bo}$=1,2,3,4,5,6. Taking the InAs lattice constant (0.60583 nm), this corresponds to dot heights from 0.6 to 3 nm, dot diameters from 7.3 to 12 nm, and the inter-dot separation for 0.6 to 3.6 nm. These are, thus, rather small systems due to our computational limitations (a relatively small number of atoms, but still reaching over 100,000). However, the latter part of the paper will study a substantially larger range of possible dimensions, including larger quantum dots and separations.

Despite limited, our choice leads to $5\times 5\times 5\times 5\times 6$ = 3750 cases, which we calculate using the atomistic tight-binding model as described in detail in the Methods section. Here, we only note that each system contains over 100,000 atoms and that the calculation of each system involves approximately 750 seconds using 8 CPU-core MPI parallelization. This is thus a total of approximately 6250 CPU hours, a relatively minor task (few days of computations) even on a small (hundreds of cores) computer cluster. Nonetheless, we are in a regime in which obtaining data samples is the most time-consuming part of the computation, and the training (e.g. neural network) should be computationally far less demanding (see Methods), which is contrary to multiple other neural networks applications where the training phase is computationally expensive.^42^

**Fig. 2:**
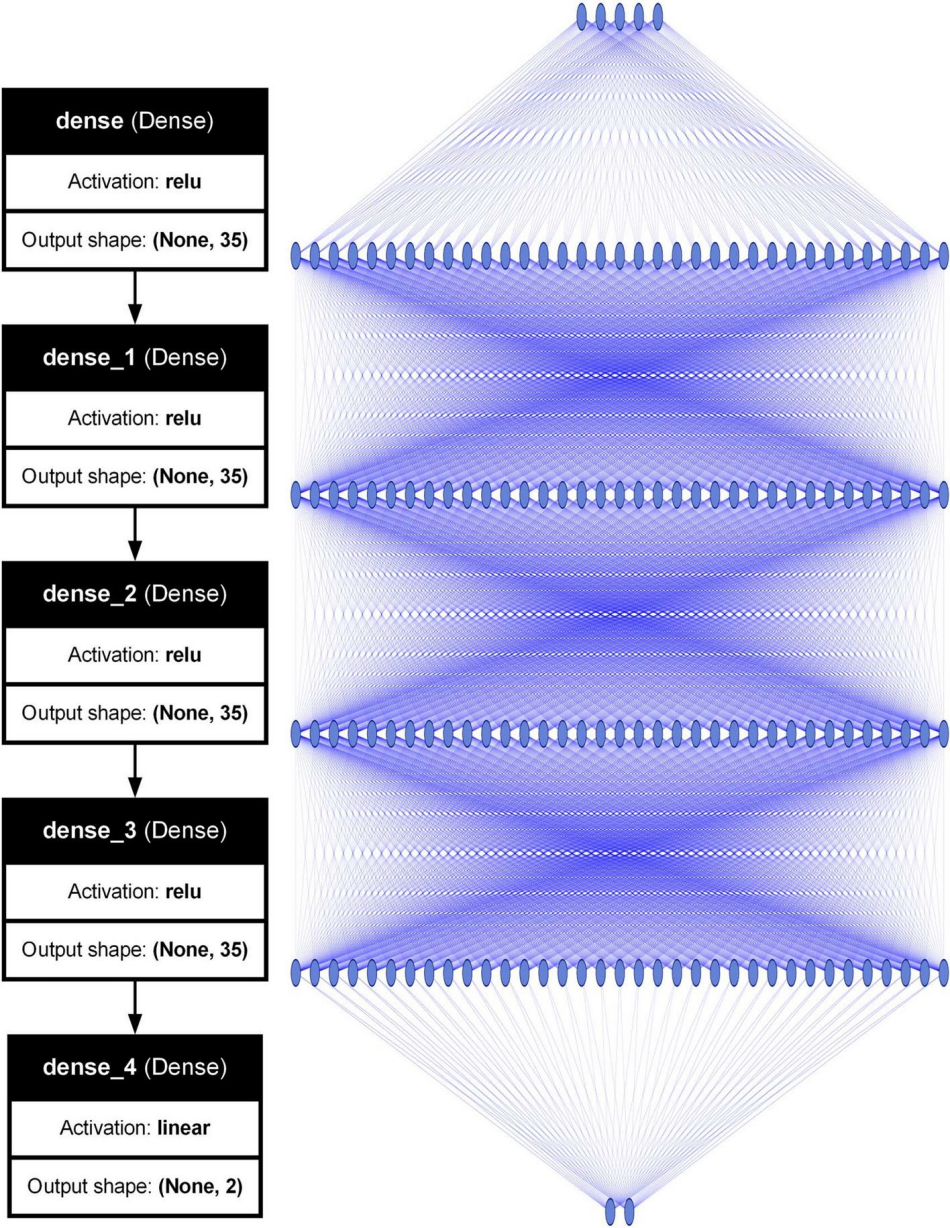
Schematics of a neural network used in this work obtained with graphviz (left) and NN-SVG (right) visualization tools. There are 5 features in the input layer and 2 labels in the output layer. The input and output layers account for 210 and 72 trainable parameters. Each hidden layer accounts for 1260 parameters, leading to a total of 4062 trainable parameters in the entire network (see Methods for more).

**Fig. 3:**
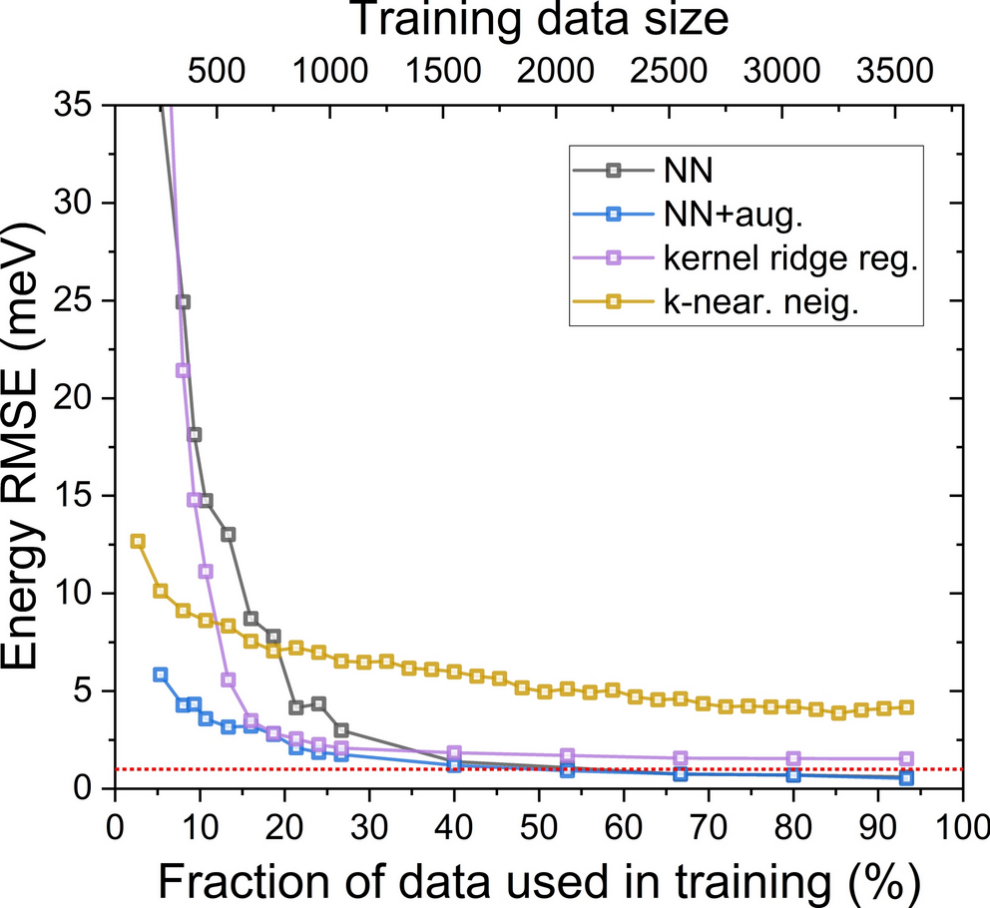
Energy RMSE (in meV’s) for different approaches discussed in the text for different fractions of a relatively small dataset involving all possible 3750 cases of double nanowire quantum dots (see the text). Dotted/red line marks 1 meV threshold. Note very different convergence of various approaches.

Once the tight-binding calculations are done, we use the obtained energies for testing several machine-learning approaches by gradually increasing the amount of data available to these methods ( Fig. 3). We compare a regression based on k-nearest neighbours, kernel ridge regression, and a small neural network (NN; see Fig. 2 and Methods). Algorithm accuracy, the vertical axis in Fig. 3, is given as root-mean-square deviation (RMSE) in meV calculated over the entire (combined) set of electron and hole energies. Except for small training sets, RMSEs for the electron and hole are comparable; similarly, the training set RMSE is similar to the validation set RMSE (there is no problem with overfitting); thus (for clarity), we present only total (combined for E1 and H1) RMSE calculated on the validation set.

The testing and validation set was obtained by randomly selecting (shuffling) samples from the entire dataset. One could argue that the choice of a random sample may affect the accuracy of learning (e.g. leading to an unbalanced training set); we will comment on that in the latter part of the work.

The RMSE, as mentioned, is calculated on a validation set, i.e., a set of samples not used in training. In Fig. 3, the validation set was identical for all approaches and was taken as 200 randomly selected samples excluded from the full (3750) dataset. We did not notice any substantial effect of a validation set size. Moreover, the validation set is relatively small to allow testing a high fraction of the entire dataset (up to a maximum number of 3550=3750-200; further increasing the validation set would lead to validation samples “leaking” into the training set).

By studying all possible cases, we found that the electron ground state energy varies from 1325.6 to 1427.7 meV and the hole from 254.7 to 366.8 meV. The energy RMSE in Fig. 3 is thus given in meV units.

Computational methods for nanostructures have certain methodological uncertainties (e.g., due to parametrization, computational box size, convergence issues, etc.). To not further add to these issues, we will aim for $\approx$1 meV accuracy of a machine learning approach. However, this is not a strict threshold, and larger RMSE values may be satisfactory for other researchers. In Fig. 3 one can notice that k-nearest neighbours method leads to a plausible (though large) RMSE even used on a small subset (10%); however, it fails to achieve near 1 meV threshold even when nearly entirely set is used for training (is saturated at 4 meV RMSE). Contrarily, kernel ridge regression fails for a very small training subset. However, it converges rapidly, reaching an RMSE of 2.5 meV at about 20%. Then, it saturates at about 1.5 meV.

Finally, neural network performance is poor for small training sets, which is a well-known phenomenon; however, close to 40% fraction of all data, it performs better than kernel ridge regression and reaches 1 meV RMSE threshold at about half ($\approx$ 2000) of samples used in training (out of all 3750). Finally, it can achieve 0.6 meV RMSE on a validation set when training is done on nearly the entire set. 0.6 meV appears to be a theoretical limit for the simple network architecture used in this work; whether this is large or small is arguable. RMSE of 0.6 meV is rather small compared to the range of possible electron and hole energies (on a 1000 meV scale); however, as the RMSE is calculated (averaged) on a validation set, it does not exclude the possibility of individual points (or group of points) having much larger deviations, as will be discussed in the latter part of the paper. On the other hand, even the simple neural network architecture may already lead to (approximately) 1 meV RMSE when using half of the data. This may already be beneficial for particularly time-consuming calculations, where getting more data points via tight-binding (or any other atomistic calculation) involves days or weeks. Finally, we comment that the neural network training process is relatively stable (Fig. 1 (c)) with no apparent sign of overfitting, although with notable fluctuations of the validation loss, likely due so much is due to a small batch size and a small validation set size (see Methods).

### Data augmentation

The neural network performance so far may be considered disappointing. As mentioned, it may be due to a relatively small dataset size. On the other hand, the k-nearest neighbour method can give a pretty decent approximation, even for a very small training set. Naturally, one could combine these two approaches to achieve a synergy effect. One way of doing so is to augment the neural network training data set with samples generated via a k-nearest neighbour. To this aim, we generate 1000 additional samples by randomly (using continuous uniform distribution) generating points in a 5-D space within the range of possible dot heights, radii, and inter-dot distances (though without the discretization, allowing for continuous values). At each point, we interpolate E1 and H1 using k-nearest neighbours trained only on the same (small) subset of data used for the network training without the augmentation. This prevents information leakage from the entire dataset to the validation set. Moreover, for the same reasons, the validation set is not augmented.

Again, our choice of data augmentation is arbitrary, and far better solutions (both regarding the interpolation and random points selection) likely exist. Nonetheless, we tested this relatively simple approach, as shown in Fig. 3, and interestingly, despite its simplicity, data augmentation works by leading to 3.5 meV RMSE for as little as 10% of the entire dataset. (Again, we note that Fig. 3, for the augmented case, shows the size of the training set before/without the augmentation.) Suppose a crude solution is satisfactory (e.g. for e1-h1, the single-particle bandgap estimation). In that case, this approach can be successful for about 1/10 of the cost of the entire calculation, with only a few additional minutes spent on training. However, we also note that the benefit of data augmentation diminishes with the increase in the training set size. Apparently, with the training size of $\approx$1000, the augmentation with an additional 1000 samples does not bring any additional information that a network could benefit from. Moreover, increasing the size of the augmentation set (2000,3000,...) does not bring any further improvement or can even detriment the result.

### More realistic, larger systems

As pointed out, systems studied so far had a limited range of possible dot dimensions. Here, we will increase the range of dot heights from 1 to 9 l.c. (i.e. 0.6 to 5.5 nm), similar with 9 possible dot radii from 6 to 14 l.c. (0.36 to 8.5 nm). Again, there are 9 possible inter-dot spacings, from 1 to 9 l.c. (0.36 to 5.5 nm). This leads to a total of $9^5$=59049 different systems. Moreover, larger dot sizes and inter-dot separations require larger computational boxes (i.e., sections of host InP nanowire) with 350,000 atoms in each tight-binding calculation.

**Fig. 4:**
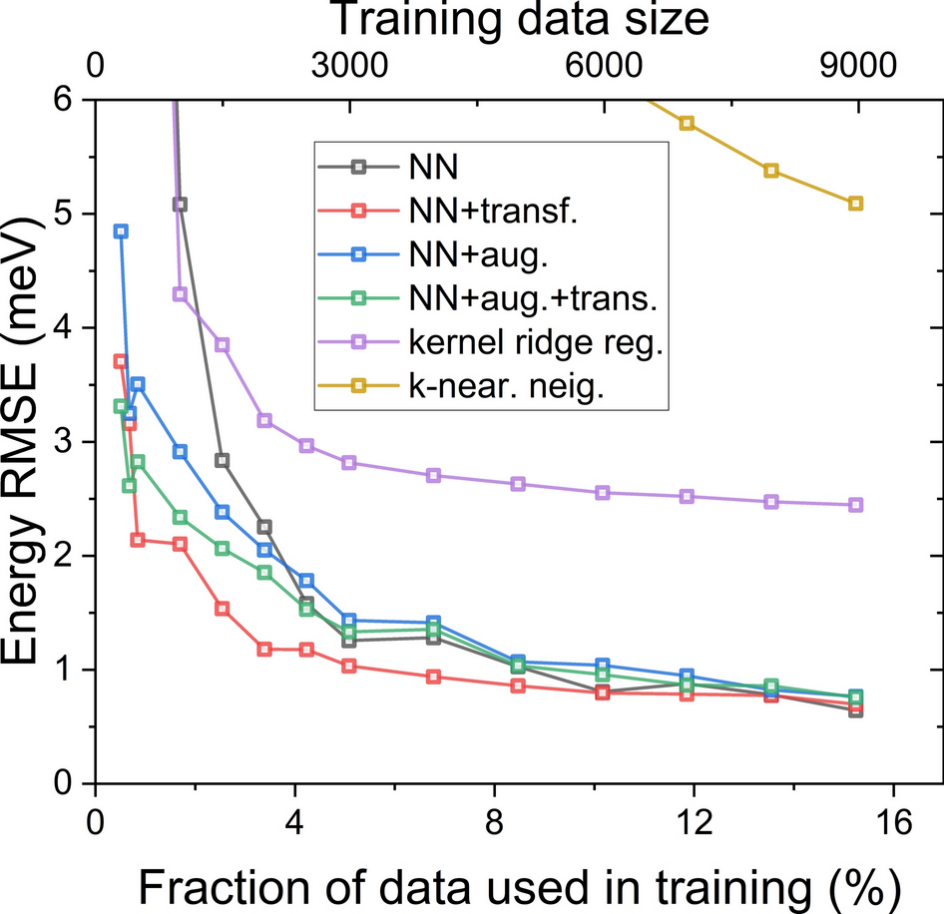
Energy RMSE (in meV’s) for different approaches discussed in the text for different fractions of a relatively large dataset involving 59049 cases of double nanowire quantum dots. Note very different convergence of various approaches and that a relatively small fraction of the entire set is needed for all neural network-based approaches to be close to the 1 meV target.

Increasing computation boxes by 3.5 factors will (in the best-case scenario) increase the computational time proportionally. However, other challenges may be related to parallelization on modern CPU architectures (with few actual processors hosting multiple cores) that will further increase computation time. Altogether, this leads to a two-order increase in computation time compared to a previously considered dataset, with a total computation time of around 0.5 million CPU hours, resulting in a formidable challenge.

In the following, we will perform a similar analysis as done previously; however, due to limited computational resources, we will not perform a complete 59049 dataset computation but will limit ourselves to at most 10,000 randomly selected cases. (Whenever we refer to these systems, it will be called as a ”larger” dataset compared to a ”small” dataset studied earlier.) We calculated energies for all that 10,000 cases and noted, that, as we perform calculations for the larger number of possible dot sizes, the energies vary in a broader range of values, i.e., the electron ground states energy varies from 1257.5 to 1410 meV, whereas the hole from 270.8 to 406.5 meV.

Fig. 4 is similar to Fig. 3, yet this time with calculations performed on a larger dataset. The only notable methodological difference is that the validation set size is 10% of the training set size (e.g. for 6000 elements in the training set, there are 600 elements in the validation set), and the augmentation was performed by always adding 2000 elements to a training set (irrespective of its original size).

Interestingly, as shown in Fig. 4, already a small fraction of data (4-8%), used for various neural network approaches, results in RMSE close to 1 meV, which is much better than kernel ridge regression, not to mention k-nearest neighbours method, which is far of the scale. Therefore, it seems that for the problem studied here, not only is the fraction of the dataset important, but the number of samples also matters. Namely, a 5% fraction of the data corresponds to $\approx$3000 samples, which is enough for the neural network to benefit from. For this reason, the data augmentation scheme brings notable benefits when augmenting the training set smaller than 2000 (3.5% of all possible cases). In contrast, it apparently contaminates (although very weakly) the training set when augmenting larger sets.

### Transfer learning

So far, we have studied two sets with a substantially different number of cases, i.e., a relatively small set with 3750 elements and a larger set with 59049 elements. We also remind the reader that the smaller set benefited (up to a certain point) from data augmentation. In contrast, the second dataset was not only more numerous, but each element took far more time to calculate (caused by a larger section of the nanowire in which it was embedded). Overall, it was relatively less time-consuming to calculate the entire small dataset. In contrast, it is challenging (depending on the available resources) to calculate even a 10% fraction of the large dataset. However, from the point of view of underlying physics, each set should share a significant part of underlying physics. In particular, the smaller dataset possesses information about smaller dot sizes and inter-dot spacing that could be beneficial in calculating larger dot heights, radii, and separations. In other words, we could think of sharing the information learned by neural networks when training on a small dataset to a larger case. We perform this via transfer learning to this aim: we took a neural network (assuming normalized inputs) trained on a smaller, computationally feasible set and applied it to a larger problem. For the large set, the network was then retrained with nearly all frozen layers, except for the output layer and the last dense layer before the output (see Methods). After the convergence (typically, 1000 epochs are more than sufficient, with no signs of overfitting and as little as a few minutes of calculation on a regular laptop), fine-tuning was performed by unfreezing all remaining layers. The results are shown in Fig. 4, with this training method outperforming all other approaches and leading close to 1 meV RMSE for as little as 4% of the large dataset.

### Testing the method

Once the training is finished, we can assess the model quality beyond simple RMSE analysis. We are not capable of performing 5D plots inspecting all 5 parameters $H_{top}$,$H_{bo}$,$R_{top}$,$R_{bo}$,$D_{top-bo}$ simultaneously; however, we can create, 2D (contour) plots by varying two variables and freezing the other three.

**Fig. 5:**
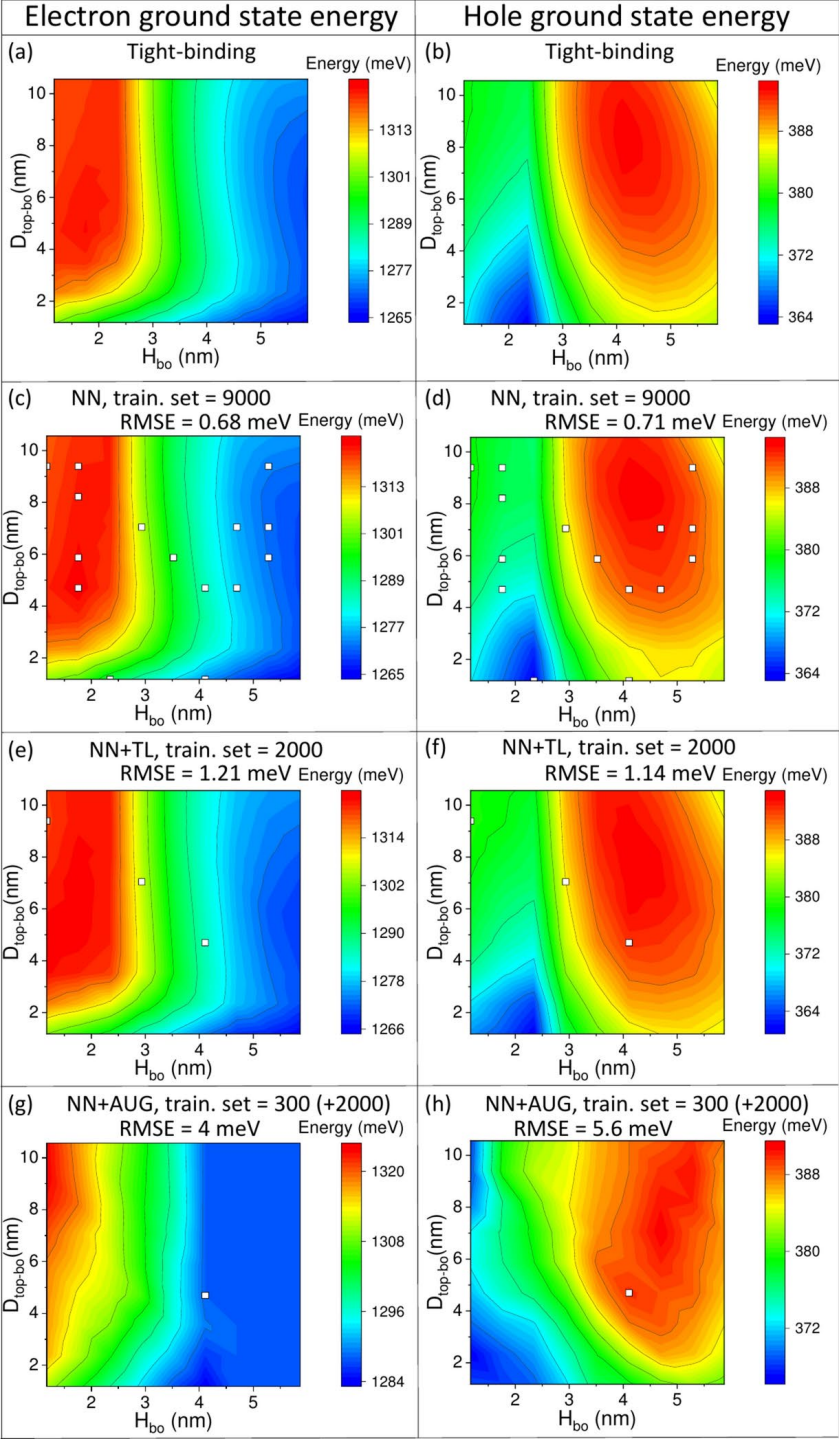
Electron (a,c,e,g) and hole (b,d,f,h) ground state energies obtained using a full tight-binding (TB) calculation (a,b); using a neural network (c,d) trained over a large number samples (9000); (e,f) using the same neural network, yet with a much lower number of samples in training (2000), and with transfer learning; (g,h) using the same neural network, yet with a very small number of samples in training (300), and data augmentation (2000), and no transfer learning. Energies are calculated as a function of inter-dot separation $D_{top-bo}$ and bottom quantum dot $H_{bo}$ height with all other parameters fixed to $H_{top}=4 l.c.\approx 2.4~nm$, $R_{top}=R_{bo}=13 l.c.\approx 7.9~nm$. The plot is a cross-section (hyperplane) of the entire search space (5-D hypercube) with 81 possible combinations of $D_{top-bo}$/$H_{bo}$, with the contour plot filling the rest of the space. Note the plot complexity despite both dots’ identical diameters ($D=15.8$ nm). White square marks points that were used in neural network training. Despite their scarcity, regions away from these points are still well reproduced by the network. Plots have been created using the contour plot feature from OriginPro 2021b (https://www.originlab.com/).

Fig. 5 shows examples of such plots calculated for different neural network models and compared with the tight-binding model. Plots in Fig. 5 were obtained by fixing the height of the bottom $H_{bo}=$2.4 nm, their radii $R_{top}=R_{bo}=$7.9 nm, while varying the height of top dot $H_{top}$, and the inter-dot separation $D_{top-bo}$. Such choice of fixed/varied parameters was largely accidental, except for $D_{top-bo}$, which is particularly often investigated in double quantum dots.

As shown in Fig. 5, neither the trend for electron nor hole energies is trivial; this is especially true in the case of the hole. The hole states in double quantum dots have complicated multi-band characters,^19^ what is reflected in their spectra. Fig. 5 also shows a very good agreement between tight-binding calculation (Fig. 5 (a)) and neural network predictions (Fig. 5 (b)). For comparison, above each plot, we show RMSE calculated separately for electron and hole on the validation set (validation set points are not shown on plots). Moreover, white squares on plots correspond to points used in training (points obtained be the augmentation are again not shown). There are 15 white squares (out of all 81) in Fig. 5 (c,d), thus not far from the 9000/59049 ratio between the training set size and the total number of points. However, there are only 3 points (out of all 81; again consistent with 2000/59049 ratio) for a case with transfer learning and $\approx$1.2 meV RMSE for both electron and hole, again corresponding to a very good quality plot (Fig. 5 (e,f)). As little as one point of the original tight-binding data points is present in Fig. 5 (g,h) (augmentation data points have random positions and thus do not lie exactly on a hyperplane studied in the figure and therefore are not shown). This is again consistent with a training/whole set size ratio, which is less than 1%, yet still, this allows (with augmentation) to reproduce electron spectra with 4 meV RMSE. At the same time, some features are lost in the hole spectra, corresponding to a larger 5.6 meV RMSE.

Overall, Fig. 5 gives an impression that the neural network can accurately reproduce tight-binding results with RMSE on the order 1 meV. At the same time, with RMSE of several meV, main spectral features still can be resolved. Whereas this is generally true, caution is needed as the colour scale and the interpolating nature of contour plots may obscure the important physical details.

**Fig. 6:**
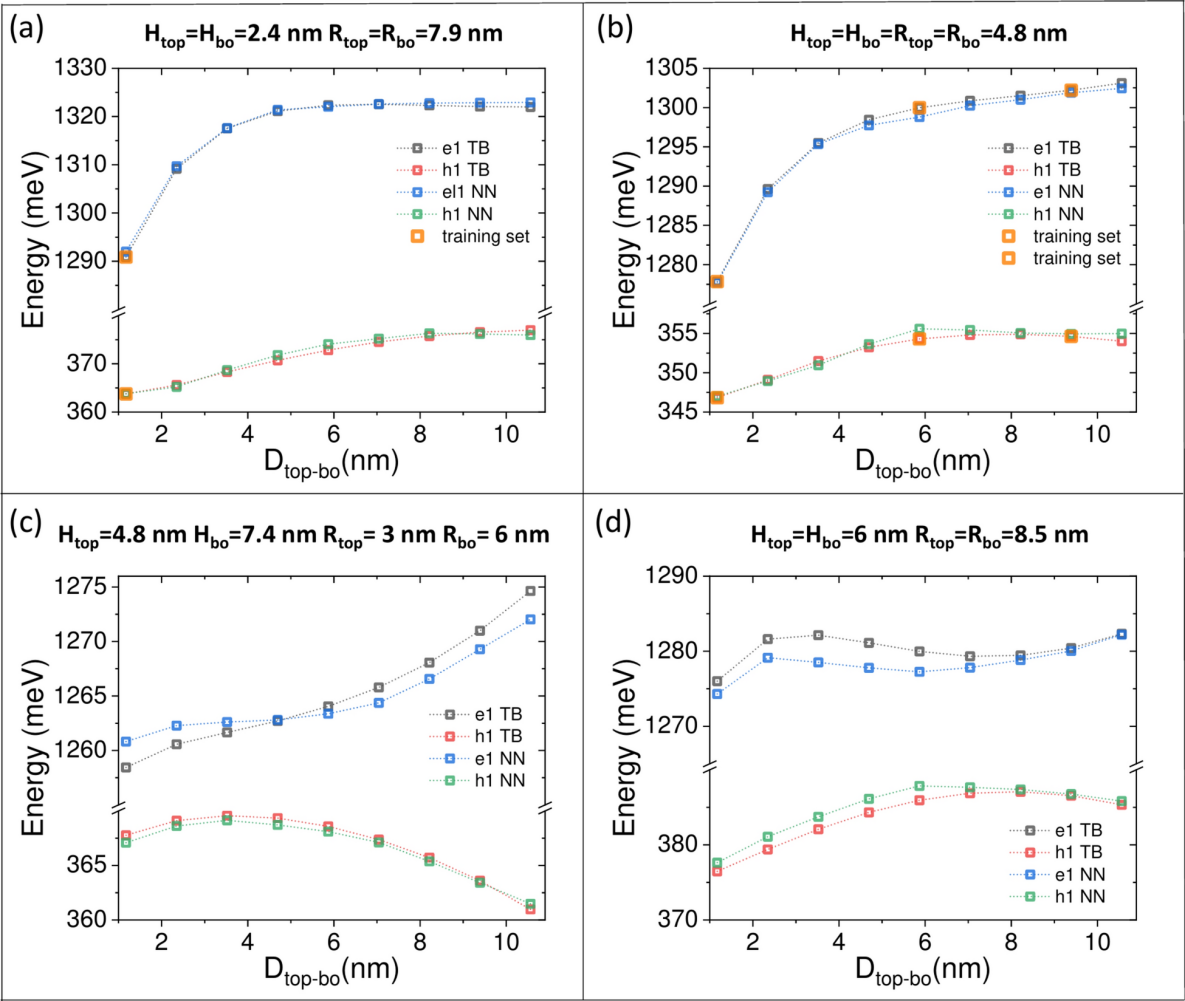
Electron (e1) and hole (h1) ground states obtained using a full tight-binding (TB) calculation as using neural network (NN), as a function of inter-dot separation $D_{top-bo}$ for different double quantum dots: (**a**) $H_{top}=H_{bo}=$2.4 nm; $R_{top}=R_{bo}=$7.9 nm; (**b**) $H_{top}=H_{bo}=R_{top}=R_{bo}=$4.8 nm; (**c**) $H_{top}=$4.8 nm, $H_{bo}=$7.3 nm, $R_{top}=$3, $R_{bo}=$6 nm; (**d**) $H_{top}=H_{bo}=$6 nm; $R_{top}=R_{bo}=$8.5 nm. Orange squares mark points were used in either neural network training or validation.

To further study the problem, Fig. 6 shows several selected systems in 1D plots, where the electron and hole energies are studied as a function of an interdot separation. Again, these plots compare the results of the full tight-binding calculations with neural network predictions (with training set size of 9000; no transfer learning or augmentation, corresponding Fig. 5 (c,d)). Consistent with the previous figure, very few training set data points (marked orange for visibility reasons) are present in the plot.

Fig. 6 (a) reveals excellent agreement of neural network prediction to the tight-binding calculation, although only one data point on the plot is explicitly used in training (however, there may be other, not-shown points, ”close” in the hyper-space to points presented on the figure).

Notably, multiple, ”original” data points on the plot do not necessarily guarantee a better agreement. One example is seen in Fig. 6 (b), where there are as many as three original (orange) data points, and the deviation between tight-binding and neural network is, in fact, the largest for 6 nm. i.e., in the exact placement of the training data point. This illustrates the natural limitation of a model and RMSE (a meticulous researcher should thus test other accuracy measures as well).

The very small deviations between full computation and machine learning prediction are consistent with RMSE < 1meV; however, RMSE, as calculated/averaged over the entire validation set, may obscure some less favourable cases. This is very well illustrated in Fig. 6 (c), and (d) when either electron [Fig. 6 (c)] or electron and hole [Fig. 6 (d)] ground state energies have few meV deviations (thus definitely more significant than RMSE of 1 meV) from the tight-binding calculation. Nonetheless, all complicated trends are still very well reproduced within a few (but not 1 meV) quantitative deviations.

The fact that the same cases are better or worse represented by the neural network may be related to the random choice of the training data. In particular, the case studied on Fig. 6 (d), corresponds to maximal values of quantum dots dimension used in the simulation. In other words, these points lie on the edge of the hypercube (formed by all possible geometries), which could be less represented in the training set than the data from within the centre of the hypercube. This illustrates the problem and the possible benefits of a more optimal (random or not) selection of training points, leading to a more balanced training set. (We note again that the list of these data points is randomly established at the beginning of the process, and the tight-binding calculation is performed only for these points.)

Finally, we note the energy dependencies shown in Fig. 6 are far from trivial, illustrating the need for calculation performed in this work. Simple modelling using the bonding/antibonding 2x2 Hamiltonian model cannot explain these trends. One reason for hole states is their multi-band character, which leads to antibonding ground states,^19,24^ and further leads to the complicated evolution of hole energies (including level crossings) as a function of the inter-dot separation. Thus, the evolution of lowest hole states typically cannot be explained by a simple model with separation between symmetric/antisymmetric states decreasing with the inter-dot separation (such a simple picture for hole would lead to hole energy evolution being a rescaled ”mirror” picture of electron ground state evolution in Fig. 6 (a)).

Importantly, the trends for electron state are also far from trivial, as shown for example in Fig. 6(c,d); this is particularly true for dots with high H/R aspect ratios or for cases where dots become close to host nanowire edges which may affect their spectra as well.

### Inverse approach

The neural network is able to reproduce tight-binding results with relatively good accuracy and minimal computational cost. In turn, this small computational cost allows solving the inverse computational problem of matching spectra to nanostructure morphological properties. One way to approach the problem is to benefit from the fact that by using a neural network, a large number of cases can be studied within minutes if not seconds. Capitalizing on this, one can use a brute-force search by testing all possible double dot morphologies to find those of desired spectra. For example, let us search for a system with electron ($e_1$) and hole ($h_1$) ground state energies equal (for whatever reason) to 1.3 and 0.3 eV correspondingly, and thus the single-particle gap ($e_1-h_1$) of 1 meV. A brute-force search (performed within seconds on a regular laptop) reveals a candidate with $e_1$=1.3215 eV and $h_1$=0.3159 eV corresponding to $H_{top}$=2.4 nm, $H_{bo}$=4.24 nm, $R_{to}$=4.24 nm, $R_{bo}$=3.6 nm, and inter-dot spacing of $D_{top-bo}=$0.6 nm. Our initial choice of $e_1/h_1$ energies was purely arbitrary and relatively far away from the energy range typically occurring in this system; therefore, we found a system with electron and hole energies shifted by 21 meV and 16 meV, respectively, thus relatively far from our target. One could choose a more physically relevant (although still arbitrary) target, e.g. $e_1$=1.33 eV and $h_1$=0.33 eV, with the same effective gap of 1 eV, but both $e_1$ and $h_1$ blue-shifted towards their more likely values. A brute-force search (again performed within seconds) reveals a candidate with $e_1$=1.3300079 eV and $h_1$=0.32998475 eV, thus within a fraction of a meV from a desired target, corresponding to $H_{top}$=4.8 nm, $H_{bo}$=3 nm, $R_{to}$=$R_{bo}$=3.6 nm, and the inter-dot spacing of $D_{top-bo}=$1.8 nm. The neural network prediction is then compared with the full tight-binding calculation for this geometry, giving $e_1$=1.3304 eV, $h_1$=0.326 eV, thus with excellent accuracy for $e_1$ and 4 meV error for $h_1$, which is plausible as compared to all other uncertainties always present in both the experimental growth and simulation. Following such algorithm one can very rapidly a find system of needed properties (withing certain error bars), verify them by a single full tight-binding calculation or confirm the impossibility of finding such a system in a given parameter space.

We also note that dots of different geometries (e.g. large radius/small height vs small radius/large heights) can have very similar electron and hole energy spectra; therefore an inverse search, such a performed here, may always be ambiguous: e.g. we found 7 other systems with different morphologies, yet with that electron and hole energies within 0.5 meV error from the desired target (at the neural network prediction level). This ambiguity may not always be undesired as it can reveal a family of systems of similar properties. In the case studied here, we found – by and inverse search – that systems with $H_{top}\approx$ 5 nm, $H_{bo}\approx$ 2.5-3nm, $R_{to}$=$R_{bo}\approx$ 4 nm, $D_{top-bo}=$1.8-2.4 nm, all have similar energy spectra. Thus, an inverse search utilizing a neural network not only works reasonably well but, in principle, can provide information useful for the growers.

Finally, we note that matching experiments with theory may involve accounting for alloying (composition intermixing) that is always present in realistic dots, which gives another dimension to the problem and strongly depends on a particular experiment. We also note that the inverse search will reveal its power when performed for alloyed systems with multiple output labels, such as excitonic binding energies, rather than single-particle energies only. We thus leave combining machine learning with the many-body studies of alloyed double nanowire quantum dots for our future work.

## Discussion

Apart from the multiple advantages discussed earlier in the text, our approach also has potential issues, as the methodology is crafted strictly for nanostructures and not for some asymptotic cases, such as bulk limits of dot and nanowire sizes. Here, we model states confined in quantum dots with effective band gaps on the order of 1 eV. It would take at least 30 nm x 30 nm x 30 nm InAs quantum ”dots” (with one million atoms in the dot region) to be even remotely close to the InAs band gap of 0.43 eV. In other words, the confinement in considered systems is very strong, and the asymptotic limit of very large dot sizes is thus far from the model applicability. This happens even at the tight-binding level, as our dots contain at most 50k atoms and not millions (the number of 350k atoms in the calculation comes mainly from the InP host nanowire). Furthermore, in some cases, we cannot (easily) simulate a larger dot; for example, the lateral size of the dot is limited by the size of the host nanowire. We cannot (in a straightforward matter) study a larger system unless we study larger nanowire sizes and so forth, as it would be unphysical (e.g., a dot bulging out of the nanowire). Similar problems happen when going to the other extreme, i.e. going to zero dot size, we would go to a regime with no confinement due to dots, but still, there would be confinement to a finite-size (finite length) host nanowire. In such a case, one would model the host nanowire states (with energies still much larger than that of bulk InP) and not bulk InP. Training the network to reproduce this limit would involve e.g. running calculations for empty host nanowires of different diameters. Such nanowires would still not have the spectra of realistic nanowires (not to mention the bulk) because really long sections ( 100 nm) of nanowires would need to be considered. In either case, modeling host nanowire states was not our goal, as the model was only crafted for double-dot systems.

However, what we believe the model should be able to reproduce is the limit of very small and very large dots, as well as, dots with substantial interdot separation. And the model does exactly that. As such, we note that we already study very small dots (very close to the physical limit) with a height of 1.2 nm (two lattice constants; 4 monolayers only), and really large dots with heights of 6 nm, as well as large interdot separations. Thus, it covers a vast majority of system sizes studied experimentally. We could, of course, go to even larger systems, but that would involve further calculations. This would, however, involve not only running those missing large sizes cases but also taking as a larger section of a host nanowire (to be able to host larger heights and dot separations), which already was mentioned when going from ,,small” to ,,large” model in the manuscript. Therefore, maintaining a consistent calculation would involve running the entire calculation from the beginning. This - in our opinion - would not bring any real benefit: the network would not improve its performance for realistic cases (as it already performs very well), and the neural network still might be not able to extrapolate to the extreme limits, as it still would not be trained for those cases.

Finally, we note that as each electron (and hole) tight-binding wavefunction (for each of thousands of ”larger”, 350k atoms cases) is represented as a vector of 7010660 ( 7 million) complex coefficients (350533 atoms x 20 spin orbitals in the sp3d5s* tight-binding parametrization). It would be extremely challenging (if not impossible) to accurately train the network to reproduce these coefficients and thus tight-binding wavefunctions with all the complicated, complex spatial phases and symmetries. However, our preliminary work suggests that it is possible to train the network to reproduce quantities calculated using those wave functions, namely Coulomb and exchange integrals (such as electron-hole attraction Coulomb matrix elements). These are indirect measures of wave function spatial distributions and symmetries and, in turn, allow for further many-body calculations of spectra excitons confined in these systems, which we will focus on in future work.

To summarize, we proposed a computational scheme combining the atomistic tight-binding method with several machine learning approaches, particularly a neural network, to efficiently calculate the lowest single-particle energies in double InAs/InP nanowire quantum dots. Such a scheme, which is, in fact, an elaborate multi-dimensional interpolation, allows for a significant reduction of the computational burden by reducing the amount of tight-binding calculation necessary to cover the entire set of possible quantum dot dimensions and separations. Our scheme is extendable for quantum dot systems involving even larger amounts of dots (e.g., multiple, stacked dots) and their geometries (e.g. deformations) in the input (as features), and/or excitonic energies etc., in the output (as labels), as there are no fundamental limitations to why our scheme could not reproduce excited states or oscillator strengths. Moreover, we successfully tested several possibilities (data augmentation, transfer learning) to increase network accuracy further. We have also pointed to potential caveats. As the network training and prediction phase is computationally very cheap, this method can solve the inverse computational problem of matching optical and energy spectra to nanostructure morphological properties. As modern-day machine learning libraries offer ready and easy-to-use tools, combining traditional (computationally heavy) atomistic tools with deep learning approaches will have large practical values for researchers designing future nanostructures that should meet certain functional traits.

## Methods

We use empirical tight-binding theory for the electron and hole states with an $\hbox {sp}^3\hbox {d}^5\hbox {s}^*$ orbital model with d-orbitals using parametrization of Jancu et al.^45^ The single-particle tight-binding Hamiltonian for a system of *N* atoms and *m* orbitals per atom can be written in the language of the second quantization as follows:^46,47^

1 $$\begin{aligned} {\hat{H}}_{TB} = \sum _{i=1}^N \sum _{\alpha =1}^{m} E_{i\alpha }c_{i\alpha }^+c_{i\alpha } + \sum _{i=1}^N \sum _{\alpha ,\beta =1}^{m} \lambda _{i\alpha ,\beta }c_{i\alpha }^+c_{i\beta } + \sum _{i=1}^N \sum _{j=1}^{near. neigh.} \sum _{\alpha ,\beta =1}^{m} t_{i\alpha ,j\beta }c_{i\alpha }^+c_{j\beta } \end{aligned}$$

where $c_{i\alpha }^+$ ($c_{i\alpha }$) is the creation (annihilation) operator of a carrier on the (spin-)orbital $\alpha$ localized on the site *i*, $E_{i\alpha }$ is the corresponding on-site (diagonal) energy, and $t_{i\alpha ,j\beta }$ describes the hopping (off-site and off-diagonal) of the particle between the orbitals on the four nearest neighbouring sites. Index *i* iterates over all atoms, whereas *j* iterates over the four nearest neighbours only. Coupling to further neighbors is thus neglected, and $\lambda _{i\alpha ,\beta }$ (on site and off-diagonal) accounts for the spin-orbit interaction following the description given by Chadi,^48^ which includes the contributions from atomic *p* orbitals. We account for strain effects using the valence force field method of Keating.^19,49–51^ Minimization of the strain energy is performed with the conjugate gradient method,^19,51^ whereas strain is incorporated into the Hamiltonian via Slater-Koster rules^52^ and the Harrison scaling law of hopping matrix elements.^53^

The InAs quantum dots are embedded in a host nanowire, which is modelled by an InP cylinder (Fig. 1 (b)) with height equal to 17.4 nm (30 InP l.c.), and diameter of 14.1 nm (24 l.c.) with 108799 atoms for the smaller case; and height of 28,2 nm (48 l.c.) and diameter of 18.8 nm (32 l.c.) with 350533 atoms for the larger case. We note that boxes are still rather small, and even larger fragments of nanowires (especially in the growth direction) should be used for the fully size-converged calculation.^54^ All the tight-binding (using MPI parallelization) calculations were performed on a 9-node computer cluster, each node equipped with two AMD EPYC 7542 32-core processors (a total of 576 cores).

For the machine learning part, we used k-nearest neighbours regression as a non-parametric supervised learning method, with the target predicted by local interpolation associated with the nearest neighbours. The algorithm uses a weighted average of the k=6 nearest neighbours, with weights equal to the inverse of their distance. Here, we take k=6; however, we did not find any strong dependence of results on k. We used kernel ridge regression as implemented in the scikit-learn library with the 6th-degree polynomial kernel. The 6th degree was chosen as a trade-off between algorithm accuracy and numerical stability of the fitting procedure. The dense neural network was implemented in Keras using sequential modes with 4 hidden layers, each containing 35 neurons with ReLu activation function, plus input and output layers, resulting in 4,062 trainable parameters and in a total of 142 neurons in dense layers (140 excluding the output layer). The fitting was performed with the Adam optimizer and a batch size of 64. The cost of training (minutes) was negligible compared to the tight-binding part of the calculation. For the transfer learning, the network was then retrained with the trainable parameter set to false for the first three layers. Then fine tuning was performed with all layers set to trainable.

## Data Availability

The datasets used and/or analysed during the current study available from the corresponding author on reasonable request.
